# No evidence for fitness signatures consistent with increasing trophic mismatch over 30 years in a population of European shag *Phalacrocorax aristotelis*

**DOI:** 10.1111/1365-2656.13376

**Published:** 2020-11-01

**Authors:** Katharine Keogan, Sue Lewis, Richard J. Howells, Mark A. Newell, Michael P. Harris, Sarah Burthe, Richard A. Phillips, Sarah Wanless, Albert B. Phillimore, Francis Daunt

**Affiliations:** ^1^ Institute of Evolutionary Biology University of Edinburgh Ashworth Laboratories Edinburgh UK; ^2^ Marine Scotland Science Marine Laboratory Aberdeen UK; ^3^ UK Centre for Ecology & Hydrology Penicuik UK; ^4^ British Antarctic Survey Natural Environmental Research Council Cambridge UK

**Keywords:** *Ammodytes marinus*, breeding phenology, environmental change, lesser sandeel, long‐term study, match–mismatch hypothesis, stabilising selection, trophic asynchrony

## Abstract

1. As temperatures rise, timing of reproduction is changing at different rates across trophic levels, potentially resulting in asynchrony between consumers and their resources. The match–mismatch hypothesis (MMH) suggests that trophic asynchrony will have negative impacts on average productivity of consumers. It is also thought to lead to selection on timing of breeding, as the most asynchronous individuals will show the greatest reductions in fitness.
2. Using a 30‐year individual‐level dataset of breeding phenology and success from a population of European shags on the Isle of May, Scotland, we tested a series of predictions consistent with the hypothesis that fitness impacts of trophic asynchrony are increasing.
3. These predictions quantified changes in average annual breeding success and strength of selection on timing of breeding, over time and in relation to rising sea surface temperature (SST) and diet composition.
4. Annual average (population) breeding success was negatively correlated with average lay date yet showed no trend over time, or in relation to increasing SST or the proportion of principal prey in the diet, as would be expected if trophic mismatch was increasing. At the individual level, we found evidence for stabilising selection and directional selection for earlier breeding, although the earliest birds were not the most productive. However, selection for earlier laying did not strengthen over time, or in relation to SST or slope of the seasonal shift in diet from principal to secondary prey. We found that the optimum lay date advanced by almost 4 weeks during the study, and that the population mean lay date tracked this shift.
5. Our results indicate that average performance correlates with absolute timing of breeding of the population, and there is selection for earlier laying at the individual level. However, we found no fitness signatures of a change in the impact of climate‐induced trophic mismatch, and evidence that shags are tracking long‐term shifts in optimum timing. This suggests that if asynchrony is present in this system, breeding success is not impacted. Our approach highlights the advantages of examining variation at both population and individual levels when assessing evidence for fitness impacts of trophic asynchrony.

## Graphical abstract

This paper incorporates phenology and breeding success data from a marine predator, and timing of availability of its prey to test for fitness signatures of increasing trophic mismatch over 30 years.

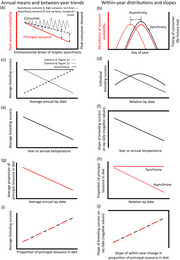

## 1 INTRODUCTION

**FIGURE 1:**
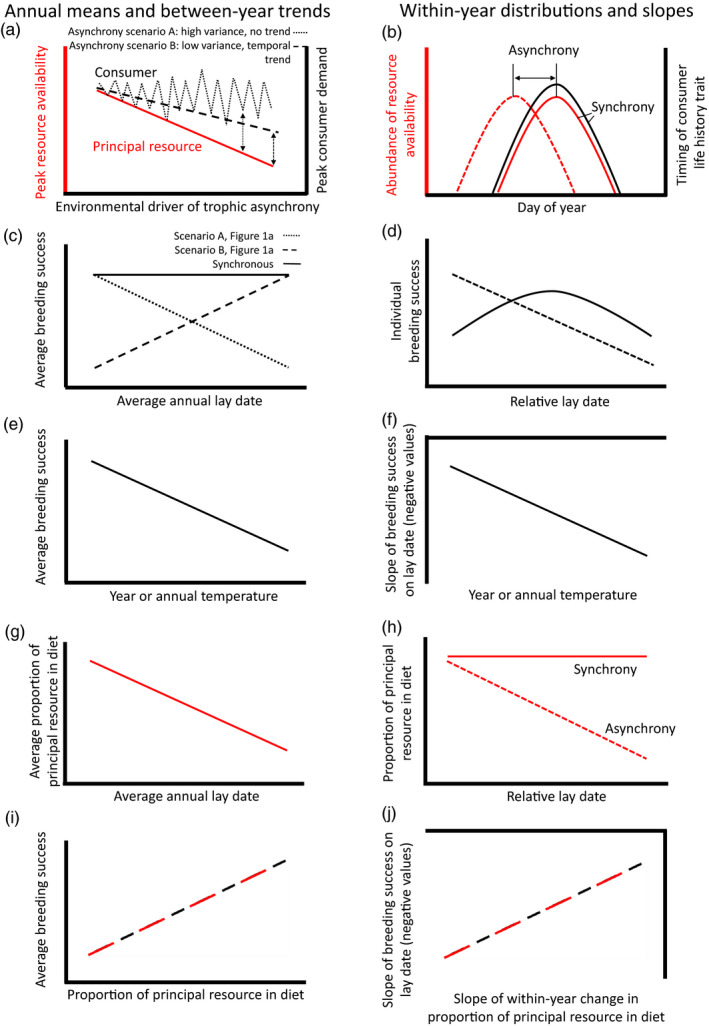
Schematic of requirements of the match–mismatch hypothesis (a, b, vertical arrows in [a] represent asynchrony) and consequences for breeding success (c, d). The impacts of asynchrony on breeding success may increase in relation to year or temperature (e, f). Asynchrony may have consequences on diet (g, h) with impacts on breeding success (i, j). Left‐hand plots (a, c, e, g and i) show expected outcomes at the population (between year) level (hereafter BSp) and right‐hand plots (b, d, f, h and j) show expected outcomes at the individual (within year) level (hereafter BSi). All asynchrony predictions are generated under the assumption that historically timings were synchronous in the average year. Red lines are representative of resource, and black lines are representative of consumer. In (a), consumer phenology is described under two scenarios, where there is no trend but substantial inter‐annual variation in timing (Scenario A), and where timing is responding linearly but more slowly than the resource (Scenario B). In (b), solid lines indicate synchronous years, and dashed lines indicate asynchronous years. In (c) negative slope corresponds to the dotted consumer line in (Scenario A in a), positive slope corresponds to dashed consumer line in (Scenario B in a) and constant slope (solid line) corresponds to synchronous consumer and resource. In (f and j), a more negative y‐value represents stronger directional selection on laying date

In recent decades, surface temperatures around the globe have risen (Stocker et al., 2013), causing the timing of seasonally recurring life‐history events, such as reproduction, to shift (Thackeray et al., 2010; Visser & Both, 2005). The plastic phenological responses of higher trophic‐level organisms to changing temperatures often appear to be weaker than those of organisms lower down the food web (Thackeray et al., 2016). Studies have shown that this difference in responsiveness could potentially lead to trophic asynchrony, whereby the timing of peak demands of consumers and availability of their resources are out of sync (Figure 1a,b; Thackeray et al., 2016; Visser et al., 2004; Visser & Gienapp, 2019). Trophic asynchrony is predicted to have negative consequences for fitness, with important implications for population dynamics (Miller‐Rushing et al., 2010; Reed, Jenouvrier, et al., 2013; Visser & Gienapp, 2019). A negative effect on demographic rates may arise because the population as a whole is less well‐matched with the availability of resources with the result that the population average breeding success or survival is reduced (Durant et al., 2007). In addition, directional selection on timing of breeding is predicted to strengthen with increasing trophic mismatch as the among‐individual variation in fitness increases (Reed, Jenouvrier, & Visser, 2013). The extent to which climate‐mediated trophic asynchrony has negatively affected demographic rates and increased selection on timing of breeding is central to understanding the population and evolutionary consequences of mismatch (Reed, Jenouvrier, & Visser, 2013; Visser & Gienapp, 2019).

In seasonal environments, temperature‐mediated trophic asynchrony may affect the relationship between timing of breeding and reproductive success in higher trophic‐level species both within and among years. Among years, average timing of breeding of consumers is predicted to advance at a slower rate than that of peak availability of their resources, driven by increasing temperature (Poloczanska et al., 2013; Thackeray, 2012). This means that all else being equal, years in which the consumer (and resource) are earliest are predicted to be the years of greatest asynchrony, leading to lower breeding success (Gienapp et al., 2014, Figure 1c‐Scenario B). However, this relationship is not predicted in cases where the consumer is not responsive to temperature and timing is subject to higher annual variation than that of the resource. In this scenario, a negative relationship between the timing of breeding and success is predicted (Gienapp et al., 2014; Figure 1c‐Scenario A). Within years, reproductive success is predicted to be highest for those individuals whose phenology is closest to the resource peak. This should result in stabilising selection in years when individuals with intermediate phenology are matched with the resource and directional selection for early breeding if the earliest breeders are matched most closely to the resource (Figure 1d; Reed, Grotan, et al., 2013). Crucially, if trophic asynchrony is increasing with recent climate change, average breeding success is expected to decline (Figure 1e) and directional selection on earlier breeding is expected to strengthen (Figure 1f; Reed, Jenouvrier, & Visser, 2013). However, there is a critical shortage of long‐term datasets on the timing of breeding of higher trophic‐level species and timing of availability of their prey, hampering the ability to test directly for the presence of trophic asynchrony and its consequences for fitness.

The match–mismatch hypothesis (MMH) was first proposed to explain changes in marine fisheries productivity (Cushing, 1990). However more recent research has mainly focussed on its prevalence in terrestrial systems (Kharouba et al., 2018; Thackeray et al., 2010; Visser & Gienapp, 2019). Our understanding of the effects of trophic decoupling on fitness in marine systems is therefore far less well‐understood than in terrestrial systems (Richardson & Poloczanska, 2008; Thackeray et al., 2010, e.g. 158 marine taxa vs. 2,918 terrestrial taxa analysed). In the marine environment, rising sea surface temperatures (SSTs) have been correlated with advances in the timing of plankton blooms (Chivers et al., 2020) and fish spawning events (Asch, 2015), and there is increasing evidence to support the MMH (Burthe et al., 2012; Régnier, Gibb, & Wright, 2017, 2019). On average, the phenology of higher trophic‐level marine groups such as seabirds has not changed over time or in response to rising SST (Keogan et al., 2018; Poloczanska et al., 2013, but see Descamps et al., 2019 for details of regional exceptions). In general the rate of change in timing of breeding in seabirds appears to be much slower than that of fish or plankton (Poloczanska et al., 2013), suggesting that seabirds are constrained in their capacity to adjust timing, potentially leaving many species at risk of becoming desynchronised with their food resources. However, it is generally quite difficult to directly test for evidence for the MMH in marine systems (Samplonius et al., 2020).

Where the MMH cannot be tested directly, an alternative approach is to quantify the effect that proxies for change in trophic asynchrony have on average breeding success, and the strength and direction of selection on timing of breeding. In seasonal breeders, two measures that are often used as proxies for change in trophic mismatch are time (i.e. years) and annual average temperature (Keogan et al., 2018). Diet composition offers an additional proxy of trophic asynchrony between consumers and their prey (Watanuki et al., 2009). In seasonal environments, consumer diet will be determined by the timing of key life‐history events in the annual cycle of the two trophic levels (Miller‐Rushing et al., 2010), and the abundance of prey in a given year (Durant et al., 2005). Accordingly, the proportion of principal prey in the diet may vary among years (Figure 1g) or within seasons (Figure 1h). Signatures consistent with the MMH at the population level include a decline in average breeding success over time (Figure 1e), with increasing temperature (Figure 1e) and when the proportion of principal prey is reduced (Figure 1i). At the individual level, fitness signatures consistent with the MMH include stronger directional selection on timing of breeding over time (Figure 1f), with increasing temperature (Figure 1f) and when average decline in proportion of principal prey within a season is steeper (Figure 1j).

In this paper, we used a long‐term dataset for a piscivorous marine top predator, the European shag *Phalacrocorax aristotelis*, to test whether average breeding success and the strength of selection on timing of breeding has changed over time or in relation to SST or diet composition. European shags are highly variable in their annual mean phenology and reproductive success within and among breeding seasons. They lay between one and four eggs at intervals of ~3 days from the first egg, with incubation taking ~5 weeks and chicks fledging ~7 weeks after they hatch. Birds in the study population on the Isle of May National Nature Reserve, south‐east Scotland (56°11ʹN, 02°33ʹW), can begin laying as early as mid‐March. The laying season lasts on average for 2.5 months but can extend to 4 months in some years. Finally, mean lay date has been shown to correlate negatively with local SST in the late winter (February/March; Frederiksen et al., 2004).

The principal prey of shags in this population is adult (‘1+ group’, individuals hatched prior to the current year) lesser sandeels *Ammodytes marinus*, with young‐of‐the‐year (‘0 group’) sandeels the second most important; together comprising 83% of chick diet during the breeding season between 1985 and 2014 (Howells et al., 2017). Sea surface temperature in the late winter (February/March) is a key driver of somatic investment and recruitment of sandeels (the number that successfully transition from 0 group to 1+ group; Arnott & Ruxton, 2002; Van Deurs et al., 2009). In populations of sandeels breeding off the east coast of Scotland, the timing of 1+ group disappearance and 0 group appearance in the water column may be dependent on temperature in the current year, which has a strong impact on the condition and therefore, behaviour of both age classes (Boulcott & Wright, 2008; Régnier et al., 2018; Rindorf et al., 2016). The 1+ group are active in the water column during spring (April/May) before burying in sandy sediments, while the 0 group become available from June onwards following metamorphosis (Wright & Bailey, 1996). Although the total proportion of lesser sandeels in the diet varies among years, shags consistently show a seasonal shift in diet from 1+ group to 0 group sandeels, following the seasonal shift in availability of each group (Howells et al., 2017). However, there is a marked variation among years in the timing of this seasonal shift, which is an important determinant of the proportions of the two prey types in the diet each year (Howells et al., 2017), although the driver of this switch is still unknown. Together these datasets provide an excellent opportunity to test whether average breeding success and selection on timing and breeding success has changed in relation to year, temperature and changes in the proportion of the principal prey in the diet.

This study has two principal aims. First, we estimate the effect of lay date on breeding success at both the population level (BSp) and individual level (BSi), addressing the effects of timing on average population breeding success, and the presence, direction and form of selection respectively. The predictions that follow are dependent on the assumption that with warming, timing of breeding of the consumer lags increasingly behind that of its main resource, increasing mismatch (Durant et al., 2007). Second, we test whether annual mean breeding success and strength of directional selection on timing of breeding have changed in relation to three proxies of asynchrony (Figure 1). If mismatch has increased over time, the fitness signatures that would be consistent with this trend are that mean annual population‐level breeding success will have declined, and strength of selection on relative lay date within a season will have increased (a) over the course of the study, (b) with increasing SST and (c) with a decline in the proportion of the principal prey, 1+ sandeels. In addition, we combine data on SST, lay date and breeding success in our study species to estimate the environmental sensitivity of selection (Chevin et al., 2015). Specifically, we test whether the optimum lay date advances with SST or over the course of the study. The optimum timing is expected to advance if the principal resource is also advancing with SST and over time. The environmental sensitivity of selection is a key parameter for predicting the ability of populations to cope with climate change (Chevin et al., 2010; Gienapp et al., 2013; Vedder et al., 2013).

## 2 MATERIALS AND METHODS

### 2.1 Data collection

#### 2.1.1 Breeding phenology and success

Breeding phenology and success were recorded for a sample of nests every year between 1987 and 2016 (range = 35–266; no data available for 1993 and 2003). Nests were monitored in 18 plots spread throughout the colony. Nests were checked every 7 days from before laying started until after the last chick had fledged. For most nesting attempts, lay date was taken to be 3 days prior to the first date that incubation was recorded. However, in some cases the number of eggs in the nest were counted on the first occasion that laying was confirmed, in which case lay date could be estimated with greater accuracy based on standard laying intervals of 3 days in this species (Potts et al., 1980). While the maximum error in lay date is an overestimate by 4 days (for a nest where laying occurred just after the previous visit), variation in accuracy across nests should be consistent within and between years, and measurement error variance will therefore be much smaller than the within‐year variance in lay date. In cases where a nesting attempt failed and a second clutch was laid at the nest, the new attempt was not included in the core analyses because lay date was not independent of the laying and failure dates of the first clutch in the same year. However, because overall breeding success of a nest (i.e. from all breeding attempts) may be impacted by the timing of the first breeding attempt, an additional analysis was included to test the effect of lay date of the first clutch on overall breeding success. We found no qualitative difference in the results between these two models and therefore our subsequent models only included first laying attempts (results for model of overall breeding success provided in Table S1.a in Appendix 1). Population counts of breeding pairs were available for each year, collected using standardised methods (Walsh et al., 1995).

#### 2.1.2 Inshore temperature data

Following Frederiksen et al. (2004), SST data were extracted for February and March in each year from http://www.bsh.de, for an area surrounding the Isle of May that overlapped shag foraging distribution in the breeding season (Bogdanova et al., 2014; bounded by *c*. 56°0ʹ–56°4ʹN, and 2°7ʹ–2°3ʹW). This period was selected because late winter temperature is a key driver of sandeel somatic investment and recruitment (the number of sandeels that successfully transition from 0 group to 1+ group; Arnott & Ruxton, 2002; Van Deurs et al., 2009). Additionally, population‐level lay date in shags is positively correlated with SST in the same time period (Frederiksen et al., 2004). We averaged the monthly records to obtain a mean late winter temperature for each year.

#### 2.1.3 Diet

Shag chicks and adults sometimes regurgitate food which can be collected during routine fieldwork, and the biomass proportions of each prey type can be estimated using standardised methods (full details in Harris & Wanless, 1991; Howells et al., 2017). Regurgitates were collected on the Isle of May during the chick‐rearing period (April–July) between 1985 and 2014 (*n* = 863; median 29 samples per year; range 4–69; Howells et al., 2017). Collection dates showed a strong positive correlation with the timing of the shag breeding season (median collection date vs. median lay date, *r* = 0.86, *n* = 25 years, 95% CI = 0.70, 0.94, *p* < 0.0001). The two most important diet types are 1+ group sandeels (70% of biomass) and 0 group sandeels (12%; Howells et al., 2017). 1+ group sandeels are replaced by 0 group sandeels over the course of the breeding season, from a predicted proportion of 1+ group of 1.00 in April to 0.24 in August (Howells et al., 2017). This shift is in line with the seasonal life history of the two age classes of sandeels. 1+ group sandeels are active in the water column in the early spring (April/May), before burying in sandy sediments for the remainder of the year, next entering the water column to spawn in midwinter. In contrast, 0 group sandeels become available from June onwards following metamorphosis from the larval stage (Régnier et al., 2018). This seasonal diet shift has been recorded in other seabirds breeding on the Isle of May (Daunt et al., 2008; Lewis et al., 2001; Wanless et al., 2004), and so it would appear that the diet of the seabird community reflects changes in availability of different sandeel age classes over the course of the breeding season. 1+ group sandeels are markedly larger than 0 group sandeels, and have a higher energy density (Harris et al., 2008; Wanless et al., 2004). We therefore predict that average breeding success will be higher when the proportion of 1+ group sandeels in the diet is higher. Further, there is marked variation among years in the proportion of 1+ group sandeels in the diet (Howells et al., 2017). This is likely to be determined by the timing and gradient of the slope of the change in proportion of the two age classes of sandeel within a year, relative to the timing of the shag breeding season. Therefore, if the 1+ group are the principal diet and matching with their availability is beneficial to fitness, we predict that there will be a positive correlation between the slope of change in proportion of the two age classes within years and the strength of directional selection on timing of breeding.

### 2.2 Statistical analysis

#### 2.2.1 Environmental variation and temporal trends

We estimated the linear slope of SST change and phenological trend over time to assess overall patterns within the study system using Generalised Least Squares (GLS) in *nlme* (Pinheiro & Bates, 2000) that took into account temporal autocorrelation via an autoregressive model of order 1, AR(1) (Box et al., 1990). We estimated the linear slope of annual mean lay date regressed on SST using a linear model. Howells et al. (2017) previously demonstrated that there is no trend in the proportion of 1+ group (vs. 0 group) over the study period.

#### 2.2.2 Phenology and breeding success

We used GLMMs in a Bayesian framework using the mcmcglmm (Hadfield, 2010) r package (v 3.2.2; R Development Core Team, 2015) as a framework for examining the relationship between lay date and breeding success (Aim 1). As we did not know the initial brood size at hatching, within our dataset we defined breeding success as the proportion of chicks fledged out of the maximum number of potential fledglings at the individual nest level (adapted from Burthe et al., 2012), with the maximum brood size of shags taken to be four (Newell et al., 2015). Nest‐level breeding success is therefore defined as *n*/(4 − *n*), *n* being the number of fledglings. Breeding success (BSi) was recorded at each nest, and therefore the nest was the unit of measure, not the individual bird, since we did not have comprehensive data on individual identity for the duration of the study. We used the nest‐level estimate of breeding success in two ways: the average annual breeding success of the population (BSp) and the within‐year breeding success of an individual nest relative to the annual mean (BSi). Breeding success was modelled with binomial family errors, which we preferred to a Poisson process given that breeding success is underdispersed as compared with the Poisson expectation. Outputs of the same models assuming Poisson family errors made no qualitative difference to the results (see Appendix 2). For all models, coefficients are presented as the mean of the posterior distribution and uncertainty is presented as the 95% credible intervals (CIs).

We included three key fixed effects in all models (Table 1):

1. Annual mean lay date, which allowed us to test whether there was a linear increase or decrease in breeding success, depending on the responsiveness to temperature and inter‐annual variation in timing (Figure 1a,c);
2. Within‐year centred relative lay date as a linear effect. The within‐year centring removed the effect of between‐year variation in lay date (van de Pol & Verhulst, 2006), and the slope of BSi on relative lay date estimates the direction and strength of selection on lay date within each year (Figure 1d);
3. Relative lay date as a quadratic effect, as finding evidence of a significant quadratic term and peak within the range of data is consistent with stabilising selection with an optimum that lies within the data range (Lande & Arnold, 1983).

**TABLE 1:** The effects included in each model in this analysis. Mean lay date refers to the average annual lay date of the population, and relative lay date refers to the annual lay date of each nest relative to the mean. Response variable represents chicks fledged at the nest level. Yearc denotes year as a mean‐centred continuous term and inshore mean denotes average February/March sea surface temperature (SST) from the area surrounding the Isle of May that overlaps shag foraging distribution during the breeding season. We use superscript BSp to identity terms that estimate trends in annual mean population breeding success. We use superscript BSi to identify terms that estimate trends in individual breeding success within a year

Model name	Response variable	Fixed effects	Random effects
Core	Chicks fledged/(4 – chicks fledged)	Mean lay date^(BSp)^; relative lay date^(BSi)^; relative lay date^2(BSi)^	Random regression allowing intercept and relative lay date slope^(BSi)^ to (co)vary among years
Core (with population size)	As above	Core + population size (log)	As above
Year	As above	Core + yearc*^(BSp)^; relative lay date:yearc^(BSi)^	As above
SST−1/SST	As above	Core + yearc^(BSp)^; Inshore mean^(BSp)^; relative lay date:Inshore mean^(BSi)^	As above
Sandeel core	Proportion 1+ group sandeels (logit transformed)	Mean sample date; relative sample date	Random regression allowing intercept and linear slope to (co)vary among years
Sandeel year	As above	Yearc; relative sample date yearc	As above
Bivariate core	Chicks fledged/(4 – chicks fledged) and proportion 1+ group sandeels (logit transformed)	Mean lay date (for both)^(BSp)^; relative lay date (for both)^(BSi)^; relative lay date^2^ (breeding success only)^(BSi)^	Random regression allowing intercept and slope of both breeding success and sandeel diet to (co)vary across years

We calculated the vertex of the quadratic curve as –*b*/2*a*, where *b* is the linear slope and *a* is the quadratic slope, to estimate the date within an average year when individual fitness (in relation to lay date) was maximised. We allowed the relative slopes to vary across years by fitting the regression lines as a random effect. Equation 1 below corresponds to our core model.

(1) $$z_{\mathrm{ij}}\;=\;\hat{\mu }\;+\;{\hat{\mu }}_{i}\;+\;{\hat{\beta }}_{B}\left({\bar{x}}_{j}\right)\;+\;{\hat{\beta }}_{W}\left({\bar{x}}_{\mathrm{ij}}\;‐\;{\bar{x}}_{j}\right)\;+\;\beta_{W_{i}}\left({\bar{x}}_{\mathrm{ij}}\;‐\;{\bar{x}}_{j}\right)\;+\;e_{\mathrm{ij}},$$

where *z* is the proportion of successful offspring (i.e. *n*/(4 − *n*)) in year *i* for nest *j* on the latent scale. *μ* represents the overall grand mean and *μ_i_* represents the deviation of the BSp in year *i*. $\bar{x}$ represents mean lay date, and ${\hat{\beta }}_{B}$ estimates the long‐term temporal slope in BSp; ${\hat{\beta }}_{W}$ represents the average within‐year slope of BSi on relative timing, and ${\hat{\beta }}_{W_{i}}$ represents the deviation from the average slope in year i. *e_ij_* represents the residual term with fixed variance. Random effects were assumed to come from a normal distribution with mean = 0 and a variance that was estimated from the data.

We used the core model to estimate the slope of breeding success on lay date at the population (BSp, annual mean lay dates) and individual (BSi, within‐year relative lay dates) levels. Since population density may impact annual breeding success, we ran a model that added log‐transformed annual population size as an additional term to the core model. While the effect was negative, consistent with density dependence, it was non‐significant (see Table S1.b in Appendix 1 for summary statistics from this model).

We then considered three additional models that test the hypothesised effects that changes in the severity of asynchrony have on breeding success (Table 1), each of which build upon the core model (Aim 2).

1. Year model: we included year as a mean‐centred continuous variable, and the interaction between year and relative lay date. If mismatch has increased over time, we predict that BSp will decline and the within‐year slope (BSi) will become steeper (Figure 1e,f). In addition, we used these data to estimate whether the optimum lay date has advanced over time (B), based on an approach developed by Chevin et al. (2015; Appendix 3).
2. SST model 1: we included SST in the current year, the interaction between SST and relative lay date, and year as a mean‐centred continuous variable to detrend the analysis (Iler et al., 2016). If the timing of the shags principal resources is more sensitive to temperature than the birds and the MMH is supported, we predict that there should be a negative relationship between SST and shag BSp and the within‐year decline in BSi with relative lay date should be steeper in warmer years (Figure 1e,f). In addition, we used these data to estimate the temperature sensitivity of optimum lay date (B), based on an approach developed by Chevin et al., (2015; Appendix 3).
3. SST model 2: This model is as described for SST model 2a, with the difference that we included SST in the previous year (SST‐1). Temperatures in the previous year have an impact on the condition of 1+ group sandeels (Boulcott & Wright, 2008; Van Deurs et al., 2009), which in turn may influence the timing of key life‐history events in the current year, notably the timing of disappearance from the water column. We therefore predict that if the previous spring was warm, shag BSp should be reduced and the within‐year slope of the relationship between BSi and relative lay date should be steeper (Figure 1e,f).
4. Diet models: we used the proportion of 1+ group sandeels in the total sandeel biomass from each diet sample to test for the seasonal shift in diet between the two age groups. Only samples which contained sandeel prey were used in this model (*n* = 745, range per year = 4–69). We tested whether years where the mean date of sample collection was later had lower than average proportions of 1+ group sandeels (Figure 1g) and lower average breeding success (Figure 1i). We then estimated the slope of the proportion of 1+ group sandeels in the diet regressed on collection date in each year (Figure 1h) and tested whether there was a positive relationship between the within‐year slopes of 1+ to 0 group diet proportions and breeding success (Figure 1j). First, we considered the diet in isolation and estimated between‐year and within‐year trends. The response variable (proportion of 1+ group sandeels) was logit transformed, with 0.01 added to both the numerator and denominator of the logit function to avoid −∞ and ∞ values for proportions of 0 and 1 respectively (Collett, 2002; Warton & Hui, 2011). We included three fixed effects in this model: (a) annual mean date of sample collection, which allowed us to test whether there was any linear increase or decrease in the average proportion of 1+ group sandeels in the diet in relation to the mean date of sample collection (as in Howells et al., 2017); (b) relative date of sample collection, which was within‐year centred (van de Pol & Wright, 2009), which allowed us to consider the direction and magnitude of seasonal shifts in diet between the two age classes of sandeel within each year and (c) to test whether the strength or direction of seasonal shifts in diet changed over time, we included the interaction between year (as above) and relative date of sample collection. We also included year as a random intercept and allowed the within‐year (relative date) slope to vary among years.

In a second diet model we focused on diet as a predictor of breeding success. In this model we treated shag breeding success and proportions of 1+ group sandeels in the diet as a bivariate response, with a binomial family error for breeding success and Gaussian for sandeel diet (after logit transformation, as described above). The model terms were as outlined in the core shag model and the above sandeel model (Table 1), with the following differences. First, the effect of within‐year timing was centred around the mean lay date of shags in each year for both shags and sandeels. Second, for each random term (the among‐year variation in intercepts and the among‐year variation in the relative timing slope), we also estimated covariance (*σ*) between shag (*Sh*) and sandeel (*Sa*). This gave a 4 × 4 variance‐covariance matrix.

$$\left[\begin{array}{cccc} {\sigma^{2}}_{B_{\mathrm{sh}}} & \sigma_{B_{\mathrm{Sa}}B_{\mathrm{Sh}}} & \sigma_{W_{\mathrm{Sh}}B_{\mathrm{Sh}}} & \sigma_{W_{\mathrm{Sa}}B_{\mathrm{Sh}}}\\ \sigma_{B_{\mathrm{Sh}}B_{\mathrm{Sa}}} & {\sigma^{2}}_{B_{\mathrm{Sa}}} & \sigma_{W_{\mathrm{Sh}}B_{\mathrm{Sa}}} & \sigma_{W_{\mathrm{Sa}}B_{\mathrm{Sa}}}\\ \sigma_{B_{\mathrm{Sh}}W_{\mathrm{Sh}}} & \sigma_{B_{\mathrm{Sa}}W_{\mathrm{Sh}}} & {\sigma^{2}}_{W_{\mathrm{Sh}}} & \sigma_{W_{\mathrm{Sa}}W_{\mathrm{Sh}}}\\ \sigma_{B_{\mathrm{Sa}}W_{\mathrm{Sh}}} & \sigma_{B_{\mathrm{Sa}}W_{\mathrm{Sa}}} & \sigma_{W_{\mathrm{Sa}}W_{\mathrm{Sh}}} & {\sigma^{2}}_{W_{\mathrm{Sa}}} \end{array}\right]$$

where *B_sh_* represents mean shag breeding success in a year, *B_Sa_* represents mean 1+ group sandeel proportion in a year, *W_Sh_* represents slope of BSi on relative timing within a year and *W_Sa_* slope of sandeel proportion on relating timing within years. If mismatch with sandeels impacts on BSp, we predict $\sigma_{B_{\mathrm{Sa}}B_{\mathrm{Sh}}}>0$, and if it impacts on BSi, we predict $\sigma_{W_{\mathrm{Sa}}W_{\mathrm{Sh}}}>0$.

#### 2.2.3 Model structure

All models were run for 100,000 iterations (400,000 for the bivariate model) to allow effective sample sizes for focal parameters to reach >1,000, sampling every 10th iteration and with the first 10,000 iterations (40,000 for the bivariate model) discarded as burn‐in. Parameter‐expanded priors were used for all random terms except the residual variance, which was fixed at 1 for binomial models and inverse Wishart for Gaussian models. Trace plots of posteriors were examined to assess autocorrelation and convergence. Statistical significance of fixed effects was inferred where 95% credible intervals did not span zero.

## 3 RESULTS

### 3.1 Temporal trends

**FIGURE 2:**
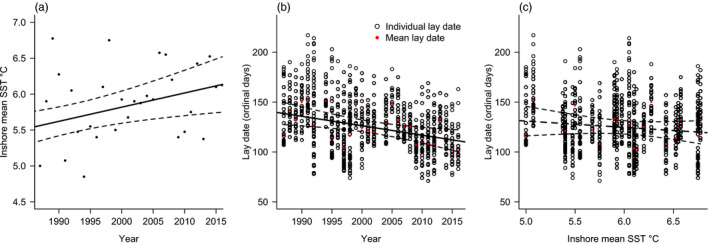
(a) sea surface temperature (SST) as a function of year, (b) lay date as a function of year and c) variation in lay date as a function of SST. Red dots depict annual mean lay dates. Black lines indicate average trends in SST (a) and lay date (b) over time, and lay date over SST (c). Solid slope estimates represent significant trends and dashed slope estimates represent insignificant trends. Ordinal day refers to number of days after January 1st, allowing for leap years. Dashed lines represent 95% confidence intervals around the slope estimate

Sea surface temperature became warmer between 1987 and 2016 (mean temperature = 5.94°C, min = 5.08°C, max = 6.78°C, *b* = 0.02 ± 0.007°C/year, *p* = 0.0085, Phi = 0.27; Figure 2a). Mean lay date became earlier during the study period (mean lay date [day of year] = 127, min = day 71 max = day 217, *b* = −0.94 ± 0.33 days/year, *p* = 0.0087, Phi = 0.15; Figure 2b). The phenological effect size of the response to SST was large but non‐significant (*b* = −6.30 ± 6.57 days/°C, *p* = 0.35; Figure 2).

### 3.2 Aim 1: Phenology and breeding success

**FIGURE 3:**
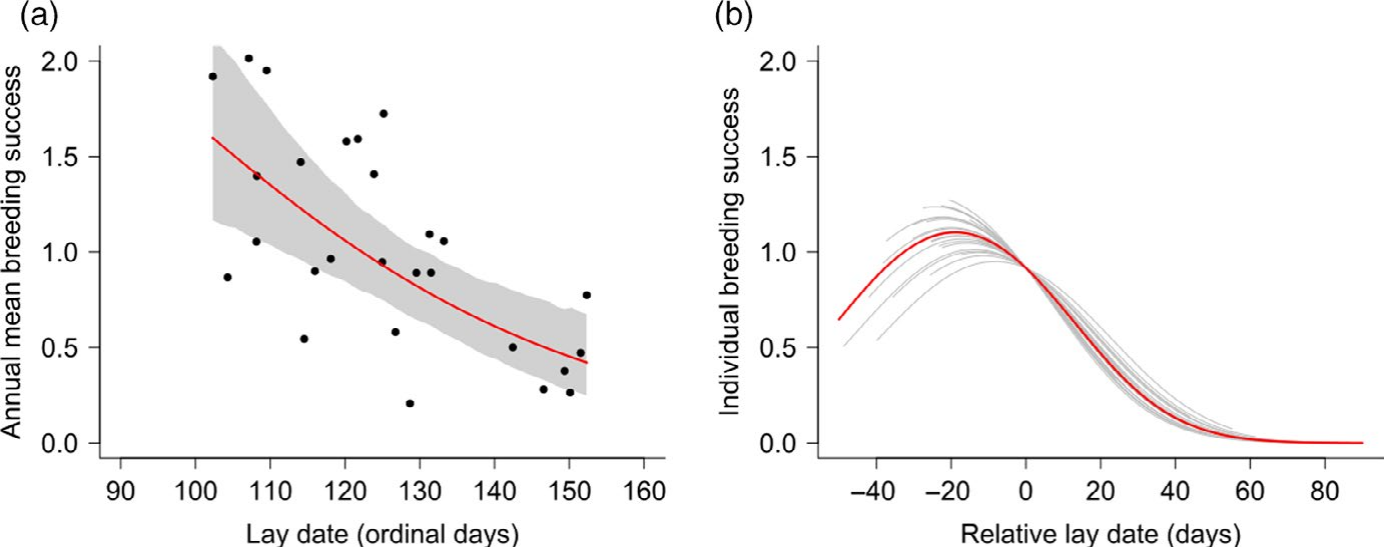
The relationship between lay date and breeding success on the data scale (a) at the between‐year level (BSp) and (b) at the within year level (BSi). Points in (a) are mean values from the data, red line corresponds to the slope across annual means estimated from the core model and estimates the change in mean fitness, and grey area corresponds to 95% credible intervals. Ordinal day refers to number of days after January 1st, allowing for leap years. Black lines in (b) correspond to best linear unbiased predictors of the within‐year slopes estimated in different years and the red line is the average within‐year slope (estimated from the fixed effects), with all coefficients taken from the core model (intercept based on the average annual lay date). See Figure S1 for a projection of these slopes on the logit scale. Solid red lines indicate significant slopes

We found that between‐years mean breeding success declined significantly with mean lay date (BSp slope = −0.035, 95% Credible Interval [CI]: −0.052, −0.016; Figure 3a; Table S2 in Appendix 1), from close to two chicks fledging in the earliest years to about 0.5 in the latest. Within years, there was a negative relationship between relative lay date and breeding success (BSi slope = −0.026, 95% CI: −0.034, −0.019; Figure 3b; Table S2 in Appendix 1) and a significant negative quadratic term (BSi slope = −0.0007, 95% CI: −0.0009, −0.0005; Figure 3b; Table S2 in Appendix 1), such that breeding success peaked in birds breeding early in the year (19.42 days prior to the annual mean [95% CI: −28.82, −11.50]), but not the earliest. A post hoc comparison of observed and predicted breeding success in relation to lay date revealed that the decline in fitness of the earliest birds described by the model was consistent with the data (Appendix Figure S3). We found no evidence that that within‐year fitness slope varied among years (core model variance = 0.0001, 95% CI: 0.0000, 0.0004; Figure 3b), meaning that we found no evidence for among year variation in the form or strength of selection.

### 3.3 Aim 2: Mismatch and breeding success

**FIGURE 4:**
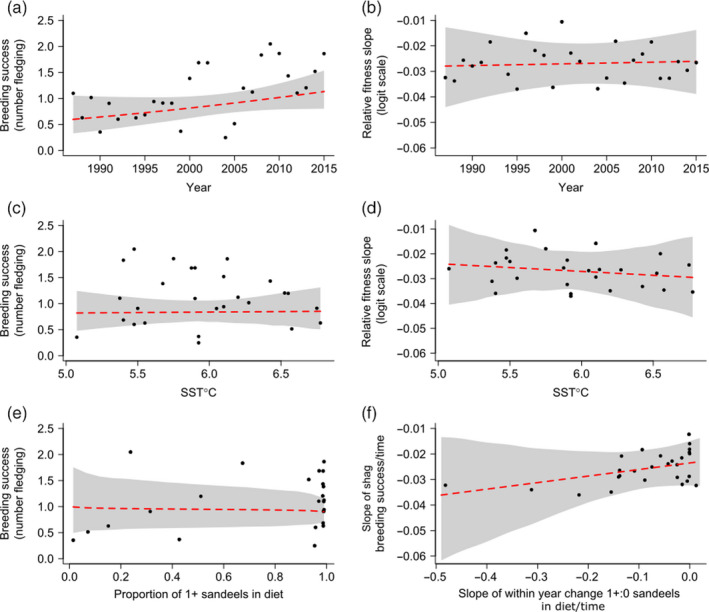
Predicted effects of year (a), sea surface temperature (SST) in the current year (c) and proportion of principal prey in diet (e) on mean population‐level breeding success (back transformed from model output). Predictions for the effects of year (b), SST in the current year (d) and the within‐year change in the proportion of principal diet (f) on the strength of directional selection on relative lay date (i.e. the slope of individual breeding success regressed on relative lay date). Black points in (a, c and e) represent annual mean estimates. Black points in (b, d and f) represent annual predictions. Red lines indicate posterior mean response and grey areas represent 95% credible intervals from the year model (a, b), the SST model (c, d), the sandeel model (e) and the bivariate model (f). For (a) and (c), model predictions are made correcting for the mean annual lay date. Dashed red lines indicate non‐significant slopes

The year model (Table S3 in Appendix 1) revealed no significant temporal decline in annual mean breeding success (BSp/year = 0.029, 95% CI = −0.008, 0.064; Figure 4a), nor steepening of the within‐year slope (BSi:year interaction = 0.00007, 95% CI: −0.0008, 0.0009; Figure 4b). However, the timing of optimum lay date advanced over the course of the study by almost 1 day per year (B = −0.96 days/year, 95% CI = −1.73, −0.17, Table S12, Appendix 3).

Spring SST in the current year had no significant effect on population‐level fitness (BSp/°C = 0.030, 95% CI = −0.592, 0.652; Figure 4c; Table S4 in Appendix 1), nor the within‐year slope (BSi relative lay date: SST slope = −0.003, 95% CI: −0.021, 0.013; Figure 4d; Table S4 in Appendix 1). Similarly, SST in the previous year had no effect on population‐level fitness (BSp/°C = −0.207, 95% CI = −0.750, 0.328; Figure S2; Table S5 in Appendix 1), nor the steepness of the within‐population slope (BSi: SST slope = 0.005, 95% CI: −0.009, 0.019). As such, warmer years neither impacted population average breeding success (BSp), nor the relative fitness of individuals breeding later or earlier than the average (BSi). Finally, there was no significant advance or delay in the timing of peak fitness for every degree of temperature rise (B = −1.81 days/°C, 95% CI = −21.98, 19.12). However, the large credible interval means that we cannot rule out the possibility that peak fitness in relation to lay date may change with temperature (Appendix 3).

**FIGURE 5:**
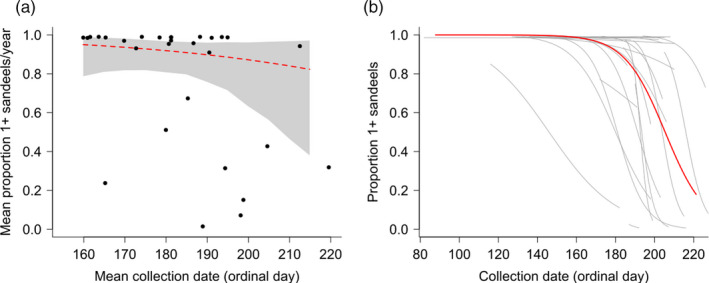
Between‐year (a) and within‐year (b) proportions of sandeels in the diet of shags during the chick‐rearing period. In (a), each point represents a yearly mean of the proportion of 1+ group sandeels in the total sandeel biomass and mean date of sample collection in that year (Ordinal day). The red line is the estimate from the core model of the change in diet proportion with mean lay date and back‐transformed from the logit scale, the grey area corresponds to 95% credible intervals. In (b), within‐year changes are represented with grey lines and the average within‐year slope across all years with a red line; all from the core model. Dashed red line indicates non‐significant slope estimate, and solid red line indicates significant slope estimate

The proportion of 1+ group sandeels in the diet varied significantly among years (variance = 6.46, 95% CI = 3.35, 11.24, Figure 5a; Table S6 in Appendix 1). However, this proportion was not correlated with annual mean date of sample collection as predicted if mismatch was present (slope = −0.025, 95% CI = −0.084, 0.028; Figure 5a; Table S6 in Appendix 1). Within a year, the proportion of 1+ group sandeels in the diet declined significantly throughout the season (relative slope = −0.095, 95% CI = −0.144, −0.049; Figure 5b; Table S6 in Appendix 1), and the within‐year slope varied significantly among years (variance between slopes = 0.015, 95% CI = 0.0076, 0.027; Figure 5b; Table S6 in Appendix 1). In an expanded model that included year as a continuous fixed effect and the interaction between year and relative timing, there was no change in the proportion of 1+ group sandeels across years (slope = 0.005, 95% CI = −0.126, 0.138; Table S7 in Appendix 1) or change in the within‐year slope over time (interaction coefficient = 0.004, 95% CI = −0.002, 0.010; Table S7 in Appendix 1).

There was no evidence for positive covariance between mean shag breeding success (BSp) and mean proportion of 1+ group sandeels in the diet ($\sigma_{B_{\mathrm{Sa}}B_{\mathrm{Sh}}}$ = −0.363, 95% CI = −2.735, 1.664; Figure 4e; Table S8 in Appendix 1). We also found no evidence of positive covariance in the within‐year slopes of relative breeding success (BSi) and proportion of the two sandeel age classes in diet ($\sigma_{W_{\mathrm{Sh}}W_{\mathrm{Sa}}}$ = 0.0003, 95% CI = −0.0005, 0.001, Figure 4f; Table S8 in Appendix 1).

## 4 DISCUSSION

We examined the effect of lay date on breeding success in a population of European shag, at both the population and individual levels. We found clear fitness benefits at the population level from breeding earlier in the year, and evidence that selection favours earlier (but not earliest) lay date, a relationship that did not vary significantly among years. We then tested whether annual mean breeding success and strength of selection on timing of breeding have changed in relation to three proxies of mismatch (time, temperature and principal prey proportion in the diet). There was no trend towards decreasing population mean fitness over time, in warmer years, or in years where 1+ group sandeels formed a smaller proportion of the diet. Moreover, the strength of selection did not vary among years, showed no trend over time or with SST and did not covary with the slope of the proportion of 1+ group sandeels in relation to date through the season. We therefore conclude that while timing of breeding appears to be inherently important for reproductive success, there are no fitness signatures consistent with an increase in climate‐induced trophic asynchrony or the MMH.

### 4.1 Effect of lay date on population‐level breeding success

At the population level, we found timing of breeding to be a negative correlate of fitness, which accords with previous studies (Clutton‐Brock, 1988; Frederiksen et al., 2004). This result is in keeping with the MMH in the situation where the consumer is showing no response to temperature and marked inter‐annual variation, as shown in Scenario A in Figure 1a. In this scenario, better matching of peak demands and availability of resources in earlier years has a positive effect on fitness. Improved synchrony may enable shags to lay more eggs on average, as has been found in populations on Sklinna and Røst, Norway (Lorentsen et al., 2015), or to have fewer losses during incubation or chick‐rearing in comparison to late years, as has been found previously on the Isle of May (Daunt et al., 2007; Frederiksen et al., 2004). However, alternative explanations could produce the same inter‐annual patterns. For example, carry‐over effects from winter conditions on surviving individuals may determine annual variation in pre‐laying conditions and, in turn, annual timing of breeding and breeding success (Daunt et al., 2014).

### 4.2 Effect of lay date on individual‐level breeding success (selection)

At the individual level, pairs breeding towards the end of the season were less successful than earlier conspecifics in all years, a result echoed in many other studies of breeding phenology (Ramirez et al., 2016; Smiley & Emmerson, 2016; Sorensen et al., 2009; Verhulst & Nilsson, 2008). The observed relationship on the Isle of May was consistent with stabilising selection around an optimum lay date that is around 19 days earlier than the annual population mean. This means that in all years, it was disadvantageous to be among the very earliest breeders, with fitness reaching a peak before declining again for the remainder of the season. The observed nonlinear trend in breeding success could result from timing lay date relative to the peak availability of resources, in keeping with the importance of trophic synchrony on fitness. There may be energetic consequences of breeding prior to or after the peak availability of resources, resulting in reduced egg production or offspring survival (Perrins, 1970; Stevenson & Bryant, 2000). Later‐than‐optimal average lay dates have been predicted by theory even in the absence of directional environmental change (Price et al., 1988). They may be a result of adaptive asynchrony, whereby the fitness benefits of matching with the peak timing of a resource are outweighed by fitness costs (Lof et al., 2012; Visser et al., 2011). Alternative explanations could also generate a negative relationship between timing of breeding and breeding success within a year. Very early breeders may be more vulnerable to factors such as increased predation risk or poor weather conditions, and late breeders may have lower foraging efficiency during winter (Daunt et al., 2014), potentially associated with migration strategy in this partially migratory population (Grist et al., 2017) or may respond less effectively to parasite burdens (Granroth‐Wilding et al., 2014; Hicks et al., 2018; Reed et al., 2008) or lower quality nest sites (Aebischer, 1985; Newell et al., 2015). Later breeders may be of lower average intrinsic quality, and therefore require longer to reach breeding condition. Later breeders are also generally younger and less experienced, with lower breeding success than more experienced breeders (Daunt et al., 2007; Potts et al., 1980). Crucially, the relationship between timing of breeding and breeding success did not vary significantly between years. There was marked an inter‐annual variation in environmental covariates, on which basis we infer that the timing and availability of resources is likely to have varied among years. As such, it appears that these alternative explanations are more likely than trophic mismatch to explain the persistent tendency for the earliest breeding birds to fledge most young.

### 4.3 Evidence for signatures of changing trophic MMH at the population level

Despite inter‐annual variation in breeding success, there was no evidence that it had declined linearly over time, with SST, or with a reduced proportion of principal prey in the diet. That breeding success has not declined over time suggests that perhaps sandeels are not adjusting their phenology at a faster rate over time than shags. This is contrary to what is suggested by multitrophic‐level phenological studies on other marine systems (Poloczanska et al., 2013). However, we do find that the optimum lay date has advanced by 0.96 days per year or approximately 4 weeks over the course of this study, and one explanation for this is that the principal prey resource has advanced over time. Such an advance would only be consistent with increasing mismatch if the consumer was shifting its timing by less than this. However, the advance in shag lay date is remarkably similar to this (−0.94 days/year) and implies that the shags are tracking optimum timing (Chevin et al., 2010), similar to results for great tits *Parus major* (Gienapp et al., 2013; Vedder et al., 2013). Based on the relatively slow life history of shags and findings from other avian systems (Charmantier & Gienapp, 2014), we suggest that this advance in shag lay date is likely due to a plastic response to an unknown environmental driver.

Sandeel phenology is likely to respond to conditions in the North Sea (Boulcott & Wright, 2008; Wright et al., 2017). Some of these conditions have shown a systematic trend over the course of the study, notably SST. However, although SST has been shown to predict sandeel phenology in a controlled environment (Boulcott & Wright, 2008), SST did not correlate with shag population‐level breeding success in this study. This suggests that shags may respond to other climatic variables, or to temperature cues at a different point in the season to sandeels, or, that sandeel availability is not an important determinant of shag breeding success. Thus, it may be that phenology of the very localised sandeel populations that shags forage on, inshore of the Isle of May, is driven by factors that we could not quantify in this study or are uncorrelated with February/March SST. It should be noted however that in this population the temperature sensitivity of the timing of optimal lay date (B, Chevin et al., 2010) may be sensitive to the temperature window used. We found no evidence of positive covariance between the proportion of 1+ group sandeels in diet samples and breeding success. Our study therefore suggests that shags do not rely on timing their breeding with the peak of availability of a single prey species. Abundance of prey may be sufficient rendering it unnecessary to coincide with a peak (Durant et al., 2005), or perhaps no clear food peak is apparent. In fact, while Isle of May shag adults do feed their chicks largely on 1+ group sandeels during the chick‐rearing period (Howells et al., 2017), they have adopted a more generalist diet in recent years (Howells et al., 2017, 2018). This may therefore serve to buffer the negative impacts of asynchrony with respect to 1+ group sandeels to maximise breeding success. Further, shags have actually advanced their lay date over time, suggesting that they may be unusual among seabirds (Keogan et al., 2018) in keeping up with the general trend towards earlier spawning of principal prey, that is, mismatch may not be present in this population. Currently phenology and abundance data do not exist at the scale required to fully examine whether trophic asynchrony across multiple prey species is present and affects breeding success of this population of shags.

Given the absence of a temporal or environmental trend, one possibility is that if asynchrony does impact on breeding success then the driver of asynchrony may not be climate‐change induced. If asynchrony is present, it may instead be driven by inter‐annual variability in environmental conditions that are largely independent of the directional trend of anthropogenic climate change (Youngflesh et al., 2017). Alternatively, reduced breeding success in later years may be unrelated to asynchrony with prey. In Adélie penguins *Pygoscelis adeliae*, timing of breeding at the population level exhibits patterns that are consistent with inherent stochasticity unrelated to measured environmental conditions, instead being embedded in the species’ breeding behaviour (Youngflesh et al., 2018). Youngflesh et al. suggest that stochastic phenology exhibited by Adélie penguins may be reinforced by their synchronous breeding behaviour, as these birds use cues from conspecifics as an indicator of when to lay. Compared with other seabirds, shags and other members of Phalacrocoracidae show especially high levels of inter‐annual variability in the mean lay date (Keogan et al., 2018), of which the drivers are not currently fully understood. Although shags are much more variable in timing of breeding within a year than Adélie penguins and other synchronous breeders (Reed et al., 2006), information transfer is possible (Evans et al., 2019), since shags set up nesting territories several weeks prior to laying.

### 4.4 Evidence for signatures of trophic MMH at the individual level

We found no evidence that the strength of linear selection on lay date varied over time, with temperature or with within‐season changes in prey availability. This is contrary to other published studies of selection on lay date, where changes in strength of selection across a variety of groups have been observed both over time, and attributed to climate‐induced changes in environmental conditions (Gienapp et al., 2013; Marrot et al., 2018; Reed et al., 2009). Thus, either the MMH may not be supported by this system, or a change in asynchrony may not be occurring at a sufficient rate to result in a detectable change in strength of selection over time and in relation to temperature or diet. Other mechanisms such as age, parasite load and nest site quality may drive selection on lay date and act consistently among years. Alternatively, any negative consequences of potential trophic mismatch may be observed either before the fledging stage, for example, between egg laying and hatching (Villemereuil et al., 2019), or at a later point in life, for example, on post‐fledging survival and reproduction of offspring throughout their lives, all periods for which we did not have data.

## 5 CONCLUSIONS

Our study supports the widespread finding that timing of breeding correlates with both breeding success at the population level and individual fitness, which is why it is extensively used to quantify the extent to which organisms respond to environmental change (Visser & Gienapp, 2019). Differential rates of phenological change across trophic levels will result in peak energy availability of the resource and the requirements of the consumer becoming asynchronous (Thackeray et al., 2010). However, there is limited evidence that if asynchrony is present, it has negative consequences for consumer fitness (Durant et al., 2007; Visser & Both, 2005; this study). There are several possible reasons why the prevalence of climate‐induced phenological mismatch has rarely been demonstrated. Firstly, the abundance of prey may outweigh the importance of being aligned with the resource peak (Durant et al., 2005). Secondly, many species at higher latitudes are trophic generalists, including shags in this population, and they may shift prey or adopt a broader diet if they miss the peak of principal prey (Howells et al., 2017). Thirdly, it may be that consumers can track prey, and our results suggest this may be the case in Isle of May shags. Finally, the fitness consequences associated with mismatch may impact different life‐history stages, rather than simply impacting upon the number of chicks to fledge the nest. To our knowledge, no marine study (and only one terrestrial system Charmantier et al., 2008; Reed, Jenouvrier, & Visser, 2013) has used timing and abundance of a full suite of potential prey species coupled with information on both adult and offspring consumer phenology, growth, survival and recruitment, and environmental variables that drive their interactions. Such analyses are urgently needed for us to fully understand the causes and consequences of changes in food web dynamics, and to predict how such systems will respond to future environmental change.

## AUTHORS' CONTRIBUTIONS

A.B.P., F.D., S.L. and K.K. conceived the ideas and designed the methodology; F.D., S.B., M.A.N., R.J.H., S.W. and M.P.H. collected and processed the data; K.K. analysed the data with support from A.B.P.; K.K. led the writing of the manuscript, with A.B.P. and F.D. making substantial contributions. All the authors contributed critically to the drafts and gave final approval for publication.

## Supporting information

Supplementary Material

## Data Availability

Data used for this publication are available from the Environmental Information Data Centre: https://doi.org/10.5285/6231bd5b‐ee2d‐4cca‐a9ef‐88006ffa4976 (Keogan et al., 2020).
