# Supplementary material for: No evidence for fitness signatures consistent with increasing trophic mismatch over 30 years in a population of European shag Phalacrocorax aristotelis
Source: J Anim Ecol. 2020 Nov 1;90(2):432–46. doi: 10.1111/1365-2656.13376 (PMC7894563; doi:10.1111/1365-2656.13376)
Supplement: Supplementary file 1 — Supplementary Material [file JANE-90-432-s001.docx]

# Appendix 1: Supporting tables and figures

**Table S1.a** Coefficients (and 95% CIs) from core model that examines the relationship between lay date and breeding success from all attempts between annual population means (superscript p) and within years among individuals (superscript i). Significant terms highlighted in bold.

| **Shags - Core Model** | Coefficient/Variance (Median and CI) | Effective sample size |
| --- | --- | --- |
| **Fixed Terms** |  |  |
| Intercept | **3.247 (0.926 - 5.413)** | 8489 |
| Mean lay date^p^ | **-0.035 (-0.053 - -0.017)** | 8399 |
| Relative lay date^i^ | **-0.034 (-0.041 - -0.029)** | 4555 |
| Relative lay date (quadratic)^i^ | **-0.0005 (-0.0007 - -0.0003)** | 1542 |
| **Random Terms** |  |  |
| (Intercept):(Intercept).year | **0.45 (0.226 - 0.819)** | 4096 |
| relative:(Intercept).year | 0.002 (-0.002 - 0.008) | 2239 |
| relative:relative.year | 0.00004 (0 - 0.0002) | 2409 |

**Table S1.b** Coefficients (and 95% CIs) from core model that examines the relationship between lay date and breeding success from first attempts between annual population means (superscript p) and within years among individuals (superscript i), with log-transformed population size included. Significant terms highlighted in bold.

| **Shags - Core Model** | Coefficient/Variance (Median and CI) | Effective sample size |
| --- | --- | --- |
| **Fixed Terms** |  |  |
| Intercept | **3.774 (0.001 – 7.060)** | 8581 |
| Mean lay date^p^ | **-0.034 (-0.053 - -0.016)** | 8215 |
| Relative lay date^i^ | **-0.026 (-0.034 - -0.019)** | 5097 |
| Relative lay date (quadratic)^i^ | **-0.0007 (-0.0009 - -0.0005)** | 1416 |
| Population size (log transformed) | -0.112 (-0.607 – 0.362) | 8410 |
| **Random Terms** |  |  |
| (Intercept):(Intercept).year | **0.497 (0.22 - 0.857)** | 2465 |
| relative:(Intercept).year | 0.003 (-0.003 - 0.009) | 3122 |
| relative:relative.year | 0.0001 (0 – 0.0004) | 2223 |

**Table S2.** Coefficients (and 95% CIs) from core model that tests predictors of variation in breeding success from the first laying attempt between annual population means (superscript p) and within years among individuals (superscript i). Significant terms highlighted in bold.

| **Shags - Core Model** | Coefficient/Variance (Median and CI) | Effective sample size |
| --- | --- | --- |
| **Fixed Terms** |  |  |
| Intercept | **3.124 (0.781 - 5.387)** | 8166 |
| Mean lay date^p^ | **-0.035 (-0.052 - -0.016)** | 8162 |
| Relative lay date^i^ | **-0.026 (-0.034 - -0.019)** | 5150 |
| Relative lay date (quadratic)^i^ | **-0.0007 (-0.0009 - -0.0005)** | 1284 |
| **Random Terms** |  |  |
| (Intercept):(Intercept).year | **0.454 (0.21 - 0.83)** | 2827 |
| relative:(Intercept).year | 0.002 (-0.003 - 0.008) | 3586 |
| relative:relative.year | 0.0001 (0 - 0.0004) | 1983 |

**Table S3.** Coefficients (and 95% CIs) from the year model that tests predictors of variation in breeding success between annual population means (superscript p) and within years among individuals (superscript i). Significant terms highlighted in bold.

| **Shags - Year Model** | Coefficient/Variance (Median and CI) | Effective sample size |
| --- | --- | --- |
| *Fixed Terms* |  |  |
| Intercept | 1.951 (-0.673 - 4.546) | 7754 |
| Mean lay date^p^ | **-0.026 (-0.047 - -0.007)** | 7738 |
| Relative lay date^i^ | **-0.027 (-0.035 - -0.02)** | 4798 |
| Relative lay date (quadratic)^i^ | **-0.0007 (-0.0009 - -0.0005)** | 1369 |
| Year (mean centred)^p^ | 0.029 (-0.008 - 0.064) | 8518 |
| Relative lay date:Year (mean centred)^i^ | 0.00007 (-0.0008 - 0.0009) | 5699 |
| *Random Terms* |  |  |
| (Intercept):(Intercept).year | **0.456 (0.196 - 0.794)** | 2664 |
| relative:(Intercept).year | 0.003 (-0.002 - 0.01) | 2839 |
| relative:relative.year | 0.0002 (0 – 0.0004) | 2374 |

**Table S4.** Coefficients (and 95% CIs) from the same year sea surface temperature model that tests predictors of variation in breeding success between annual population means (superscript p) and within years among individuals (superscript i). Significant terms highlighted in bold.

| **Shags - SST Model** | Coefficient/Variance (Median and CI) | Effective sample size |
| --- | --- | --- |
| *Fixed Terms* |  |  |
| Intercept | 1.807 (-2.421 - 6.168) | 8406 |
| Mean lay date^p^ | **-0.026 (-0.047 - -0.006)** | 8443 |
| Relative lay date^i^ | -0.008 (-0.108 - 0.091) | 3991 |
| Relative lay date (quadratic)^i^ | **-0.0007 (-0.0009 - -0.0005)** | 1456 |
| Year (mean centred) | 0.028 (-0.008 - 0.063) | 8384 |
| Inshore SST (present)^p^ | 0.03 (-0.592 - 0.652) | 9000 |
| Inshore SST (present): Relative lay date^i^ | -0.003 (-0.021 - 0.013) | 4084 |
| *Random Terms* |  |  |
| (Intercept):(Intercept).year | **0.447 (0.209 - 0.841)** | 2594 |
| relative:(Intercept).year | 0.003 (-0.003 - 0.01) | 3357 |
| relative:relative.year | 0.0001 (0 – 0.0004) | 2020 |

**Table S5.** Coefficients (and 95% CIs) from past sea surface temperature model that tests predictors of variation in breeding success between annual population means (superscript p) and within years among individuals (superscript i). Significant terms highlighted in bold.

| **Shags - SST -1 Model** | Coefficient/Variance (Median and CI) | Effective sample size |
| --- | --- | --- |
| *Fixed Terms* |  |  |
| Intercept | 3.33 (-1.204 - 7.54) | 8508 |
| Mean lay date^p^ | **-0.027 (-0.047 - -0.006)** | 8116 |
| Relative lay date^i^ | -0.054 (-0.141 - 0.024) | 5200 |
| Relative lay date (quadratic)^i^ | **-0.0006 (-0.0009 - -0.0005)** | 1310 |
| Year (mean centred) | 0.03 (-0.005 - 0.065) | 7987 |
| Inshore SST (past)^p^ | -0.207 (-0.75 - 0.328) | 9000 |
| Inshore SST (past): Relative lay date^i^ | 0.005 (-0.009 - 0.019) | 5389 |
| *Random Terms* |  |  |
| (Intercept):(Intercept).year | **0.431 (0.203 - 0.809)** | 2998 |
| relative:(Intercept).year | 0.003 (-0.003 - 0.01) | 2436 |
| relative:relative.year | 0.0001 (0 – 0.0004) | 2179 |

**Table S6.** Coefficients (and 95% CIs) from core sandeel model (proportion of 1+: 0 group sandeels) that tests changes in proportion of 1+ sandeels between annual sample means (superscript p) and within years among samples (superscript i). Significant terms highlighted in bold.

| **Sandeels - Core 1+:0 group** | Coefficient/Variance (Median and CI) | Effective sample size |
| --- | --- | --- |
| *Fixed Terms* |  |  |
| Intercept | 6.983 (-2.921 - 17.744) | 5241 |
| Mean collection date^p^ | -0.025 (-0.084 - 0.028) | 5051 |
| Relative collection date^i^ | **-0.095 (-0.144 - -0.049)** | 9110 |
| *Random Terms* |  |  |
| (Intercept):(Intercept).year | **6.459 (3.351 - 11.242)** | 1757 |
| relative:(Intercept).year | **0.177 (0.036 - 0.369)** | 6006 |
| relative:relative.year | **0.0151 (0.0076 - 0.0272)** | 2872 |

**Table S7.** Coefficients (and 95% CIs) from expanded sandeel model (proportion of 1+: 0 group sandeels) that tests changes in proportion of 1+ sandeels between annual sample means (superscript p) and within years among samples (superscript i). Significant terms highlighted in bold.

| **Sandeels - Expanded 1+:0 group** | Coefficient/Variance (Median and CI) | Effective sample size |
| --- | --- | --- |
| *Fixed Terms* |  |  |
| Intercept | 9.292 (-2.384 - 21.366) | 8327 |
| Mean collection date^p^ | -0.038 (-0.106 - 0.024) | 8322 |
| Relative collection date^i^ | **-0.091 (-0.14 - -0.043)** | 9000 |
| Year (mean centred)^p^ | 0.005 (-0.126 - 0.138) | 9000 |
| Relative collection date: Year (centred)^i^ | 0.004 (-0.002 - 0.01) | 9000 |
| *Random Terms* |  |  |
| (Intercept):(Intercept).year | **6.46 (3.461 - 11.189)** | 2077 |
| relative:(Intercept).year | **0.182 (0.039 - 0.375)** | 8718 |
| relative:relative.year | **0.0151 (0.0071 - 0.0269)** | 2810 |

**Table S8.** Coefficients (and CIs) from bivariate model (chicks fledged : 1+ : 0 group sandeels) that tests the covariance between changes in proportion of chicks fledged and changes in proportion of 1+ sandeels, between annual population and sample means (superscript p) and within years among populations and samples (superscript i). Significant terms highlighted in bold.

| **Bivariate - Chicks fledged: SE1:SE0** | Coefficient/Variance (Median and CI) | Effective sample size |
| --- | --- | --- |
| *Fixed Terms* |  |  |
| Intercept - Chicks fledged | **3.151 (0.258 - 6.029)** | 6265 |
| Intercept - Proportion of sandeels | **14.996 (1.572 - 27.92)** | 611 |
| Chicks fledged:mean lay^p^ | **-0.035 (-0.058 - -0.013)** | 6236 |
| Proportion of sandeels:mean lay^p^ | **-0.0256 (-0.0334 - -0.0182)** | 550 |
| Chicks fledged:relative lay^i^ | **-0.026 (-0.033 - -0.018)** | 11463 |
| Proportion of sandeels:relative lay^i^ | **-0.085 (-0.136 - -0.034)** | 1745 |
| Relative lay date (quadratic)^i^ | **-0.0007 (-0.0009 - -0.0005)** | 1496 |
| *Random Terms* |  |  |
| Mean shag lay:mean sandeel sample^p^ | -0.363 (-2.735 - 1.664) | 1429 |
| relative shag lay:relative sandeel sample^i^ | 0.0003 (-0.0005 - 0.001) | 9595 |
| *Residual variance* |  |  |
| Residual - chicks fledged | **1.178 (0.973 - 1.4)** | 4689 |
| Residual - sandeel sample | 1 (1 - 1) | 0 |


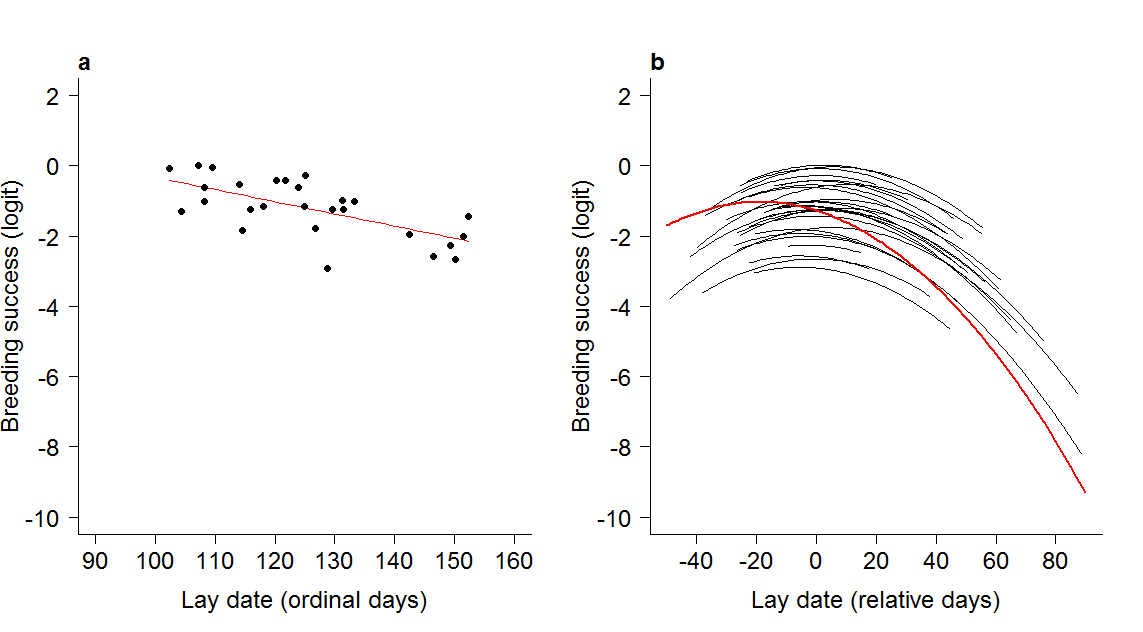


**Figure S1.** The relationship between lay date and breeding success on the logit scale (a) at the between-year level (BSp) and (b) at the within year level (BSi). Points in (a) are mean values from the data, red line corresponds to the slope across annual means estimated from the core model and estimates the change in mean fitness. Ordinal day refers to number of days after Jan 1^st^, allowing for leap years. Black lines in (b) correspond to best linear unbiased predictors of the within-year slopes estimated in different years and the red line is the average within-year slope, with all coefficients taken from the core model.


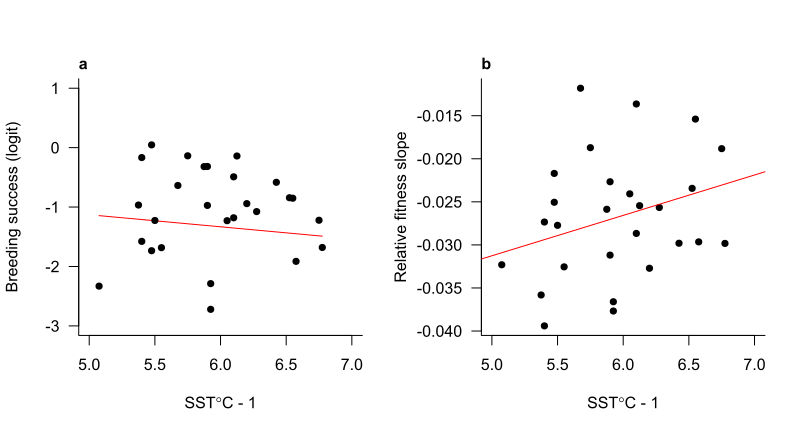


**Figure S2.** The effect of SST in the previous year (a) on breeding success (logit transformed) at the population level. Changes in strength of selection with SST in the previous year (b). Red lines indicate average response.


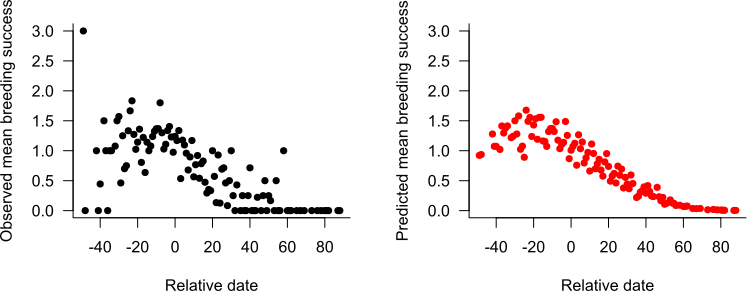


**Figure S3.** Observed values from raw data versus predicted estimates from core model of breeding success regressed on relative lay date. The mean expectation was generated via 1000 a posteriori simulations under the core model

## Appendix 2. Models with Poisson error structure

**Methods**

All models were estimated using a binomial family error structure, because breeding success was underdispersed as compared with the expectation under a Poisson process. However, to ensure there was no qualitative difference in the results, we reran each shag and co-variance (shag & sandeel) model assuming a Poisson error. In this case, the response variable was the number of chicks fledged per nest, with all fixed and random effects the same as described by equations 2 & 6 (main text) and in Table 1. Parameter expanded priors were used for all models, and the residual variance was not fixed at 1.

**Results**

There was no qualitative difference in the results under a Poisson error structure for shag (Table S9) or co-variance (Table S10) models.

**Table S9.** Key results from core models, assuming a Poisson family error structure.

|  | **As average lay date increases** | **over time** | **SST** | **SST -1** |
| --- | --- | --- | --- | --- |
| **Change in mean breeding success** | **-0.020**  **(-0.032, -0.009)** | 0.020  (-0.007, 0.045) | 0.057  (-0.404, 0.504) | -0.129  (-0.524, 0.260) |
|  |  |  |  |  |
| **Change in relative breeding success (linear)** | **-0.017**  **(-0.022, -0.012)** | 0.0002  (-0.0004, 0.0007) | -0.002  (-0.014, 0.009) | -0.004  (-0.007, 0.015) |
|  |  |  |  |  |
| **Change in relative breeding success (quadratic)** | **-0.0005**  **(-0.0007, -0.0004)** | - | - | - |

**Table S10.** Key results from co-variance model, assuming a Poisson family error structure.

|  | **1+ : 0 group** |
| --- | --- |
| **Between-year proportions** |  |
| Covariance with mean shag breeding success | -0.040  (-0.276, 0.141) |
| **Within-year proportions** |  |
| Covariance with slope of relative shag fitness | 0.000009  (-0.0002, 0.0002) |

Appendix 3. Estimating the environmental sensitivity of optimum lay date (*B*)

**Methods**

In addition to estimating the effect of various predictors (i.e. time, sSST and early vs late years) on the shape and strength of fecundity selection on lay date, we estimated the temperature sensitivity of optimum lay date (*B*). The model, based on (Chevin *et al.*, 2015), was as follows:

$\ln W_{i}\left( z \right)= \mu_{i}+\tau_{i}+\left( \beta+\zeta_{i} \right)z+ \beta_{zz}z^{2}+ \beta_{xz}x_{i}z+\beta_{x}x_{i}$ eq. S1

where $W_{i}\left( z \right)$ is the expected number of offspring, which we fit as a binomial term (see above). $\mu_{i}$ is the mean breeding success and $\tau_{i}$ is the random annual deviation in population mean breeding success in year *i*. $z$ is absolute lay date, with $\beta_{z}$ a fixed effect describing the average linear relationship between lay date and breeding success and a $\zeta_{i}$ random deviation from this slope in year *i* (i.e. fitted as a random term). The distribution of $\tau_{i}$ and $\zeta_{i}$ has a mean of 0 and the variance of these terms and the covariance between them is governed by a 2 x 2 VCV matrix that is estimated. $\beta_{zz}z^{2}$ allows for a quadratic effect of absolute timing, and the interaction between $z$ and temperature $(x)$ in year $i$ allows the optimum timing to covary with temperature. Chevin et al. did not include a main effect of temperature, $\beta_{x}x_{i}$, but we include it as otherwise the $\beta_{xz}x_{i}z$ term is sensitive to the correlation between temperature and breeding success.

In addition we run a second model that is as above but replacing mean centred SST with mean centred year.

Models were run for 100,000 iterations, removing the first 10,000 as burnin, with priors as described in main methods. For the derived quantities that we define below we obtained the mean and credible interval based on the posterior distribution.

Following equation 4 in Chevin et al. (2015) we estimated the environmental sensitivity of selection, *B*, as:

$B=-\frac{\beta_{xz}}{2\beta_{zz}}$ eq. S2

If the timing of the optimum laydate advances in years when SST is higher or is getting earlier over time we predict *B* < 0.

Following Chevin et al. (2015) we also estimated the width of the fecundity selection fitness peak, $\omega$, as:

$\omega=\sqrt{-\frac{1}{2\beta_{zz}}}$ eq. S3

**Results**

The SST model revealed a quadratic relationship between breeding success and absolute lay date (b^2^ -0.0005, 95% CI = -0.0007, -0.0003, Table S11, Appendix 3), but there was no change in the relationship between absolute lay date and breeding success as SST increased (BS absolute lay date: SST = -0.001 days°C^-1^, 95% CI = -0.02, 0.017, Table S11, Appendix 3). The SST sensitivity of selection was non-significant, implying that as SST increases the timing of the optimum does not show a significant advance or delay (B = -1.81 days°C ^-1^, 95% CI = -21.98, 19.12). The width of the fitness peak $\omega$ was 33.66 days (95% CI = 26.62, 41.46).

The year model revealed a quadratic relationship between breeding success and absolute lay date (b*^2^* = -0.0005, 95% CI = -0.0007, -0.0004, Table S12), and a significant negative relationship between absolute lay date and breeding success over time (BS absolute lay date: year = -0.001, 95% CI = -0.002, -0.0002, Table S12). Breeding success was higher in warmer years (BS year = 0.15, 95% CI = 0.05, 0.25, Table S12) and there was a significant negative interaction between absolute lay date and year (BS year = -0.001, 95% CI = -0.002, -0.0002, Table S12, Appendix 3). Inspection of the predicted relationship between absolute lay date and breeding success in each year reveals a trend for the optimum timing to become earlier and slightly higher. We find a significant delay in the timing of the optimum laydate over time, such that each year the optimum timing has become about a day earlier (B = -0.96 days year ^-1^, 95% CI = -1.73, -0.17). The width of the fitness peak $\omega$ was 31.02 days (95% CI = 25.68, 36.75).

**Table S11.** Coefficients (and 95% CIs) from the model that tests absolute lay date and SST as predictors of variation in breeding success from the first laying attempt. Significant terms highlighted in bold.

| **Shags – SST:Absolute lay date model** | Coefficient/Variance (Median and CI) | Effective sample size |
| --- | --- | --- |
| **Fixed Terms** |  |  |
| Intercept | -6.37 (-20.09 - 6.38) | 6379 |
| SST | 0.29 (-1.93 - 2.56) | 5520 |
| absolute lay date | 0.09 (-0.02 - 0.20) | 5710 |
| absolute lay date (quadratic) | **-0.0005 (-0.0007 - -0.0003)** | 1573 |
| absolute lay date:SST | -0.001 (-0.02 - 0.017) | 5213 |
| **Random Terms** |  |  |
| (Intercept):(Intercept).year | **3.66 (0.11 - 8.02)** | 2341 |
| absolute:(Intercept).year | -0.03 (-0.06 - 0.0006) | 2170 |
| absolute: absolute.year | **0.0002 (0.00001 - 0.0005)** | 2054 |

**Table S12.** Coefficients (and 95% CIs) from the model that tests absolute lay date and year as predictors of variation in breeding success from the first laying attempt. Significant terms highlighted in bold.

| **Shags – Year:Absolute lay date Model** | Coefficient/Variance (Median and CI) | Effective sample size |
| --- | --- | --- |
| **Fixed Terms** |  |  |
| Intercept | **-6.28 (-9.34 - -3.20)** | 2104 |
| year | **0.15 (0.05 - 0.25)** | 5648 |
| absolute lay date | **0.11 (0.06 - 0.16)** | 2052 |
| absolute lay date (quadratic) | **-0.0005 (-0.0007 - -0.0004)** | 1850 |
| absolute lay date:year | **-0.001 (-0.002 - -0.0002)** | 5784 |
| **Random Terms** |  |  |
| (Intercept):(Intercept).year | 1.92 (0.0000003 - 4.77) | 1221 |
| absolute:(Intercept).year | -0.016 (-0.04 - 0.0008) | 1254 |
| absolute: absolute.year | 0.0002 (0.000 - 0.0004) | 1254 |
